# First karyotype description of the Indochinese shrew *Crocidura indochinensis* Robinson et Kloss, 1922 (Mammalia, Eulipotyphla, Soricidae) from Yunnan, China

**DOI:** 10.3897/compcytogen.20.191955

**Published:** 2026-09-01

**Authors:** Jiayu Jin, Jing Dai, Jianping Ye

**Affiliations:** 1 School of life and environmental sciences, Shaoxing University, Shaoxing, China School of life and environmental sciences, Shaoxing University Shaoxing China

**Keywords:** Chromosome complement, comparative cytogenetics, *Crocidura indochinensis*, G-banding, Insectivora, karyotypes, Soricomorpha, white-toothed shrew

## Abstract

The small-sized *Crocidura horsfieldii* (Tomes, 1856) species complex, comprising *C. watasei* (Kuroda, 1924), *C. tadae* (Tokuda et Kano, 1936), *C. kurodai* (Jameson et Jones, 1977), *C. wuchihensis* (Wang, 1966), and *C. indochinensis* (Robinson et Kloss, 1922), has long been the subject of taxonomic confusion in the literature. Here, we report for the first time the karyotype of female *C. indochinensis* from Yongde Snow Mountain, based on conventional Giemsa staining and the GTG-banding technique. *C. indochinensis* has 2n = 38 and FN = 56. The autosomes consist of four metacentric or submetacentric pairs, four subtelocentric pairs, and ten acrocentric pairs, whereas the X chromosome is a medium-sized submetacentric. Our results demonstrate that the karyotype of *C. indochinensis* differs markedly in 2n and FN from those of *C. horsfieldii*, *C. watasei*, *C. tadae* and *C. kurodai*, providing further evidence for the distinct species status of *C. indochinensis* within this complex. Furthermore, comparative analysis of the G-banded chromosomes of *C. indochinensis* and *C. dsinezumi* revealed a high degree of karyotypic homology, with their karyotypes differing primarily by a single fusion/fission event and a centromeric shift or a pericentric inversion, thereby enhancing our understanding of karyotype evolution within the genus *Crocidura* Wagler, 1832.

## Introduction

The genus *Crocidura* Wagler, 1832 belongs to the subfamily Crocidurinae of the family Soricidae within the order Eulipotyphla (formerly Insectivora). Members of this genus are commonly known as white-toothed shrews, owing to the distinctive white outer layer of their teeth. It is the largest genus of mammals, comprising approximately 205 species distributed throughout the Palearctic, Oriental, and Afrotropical regions (Hutterer 2005; Burgin et al. 2018). Based on allozyme electrophoretic characters, this genus was divided into two monophyletic groups, the Palearctic–Oriental clade and the African clade (Maddalena 1990). Despite extensive studies in recent decades, the taxonomy and systematics of *Crocidura* remain uncertain and controversial. The species within the genus exhibit remarkably similar external morphology, differing mainly in body size, relative tail length and tail hairiness, which makes them extremely difficult to distinguish (Hoffmann and Lunde 2008). Therefore, in addition to morphological and phylogenetic analyses, classic chromosomal analyses serve as cytogenetic evidence for the reliable identification of *Crocidura* species.

Up to now, less than one-third of *Crocidura* species have been karyotyped using conventional Giemsa staining and various banding techniques, with diploid numbers ranging from 2n = 22 to 2n = 68 (Zima et al. 1998; Schlitter et al. 1999). Furthermore, considerable karyotypic variation has been reported among *Crocidura* species, whereas little or no variation has been observed at the intraspecific level (Maddalena and Ruedi 1994; Ruedi and Vogel 1995). Although phylogenetic analyses based on genetic sequences are the most reliable tools for species identification, these findings suggest that karyotypes, as one of the distinguishing characteristics of each species, can also provide reliable information for classification and for resolving taxonomic problems within *Crocidura*.

The small-sized *Crocidura horsfieldii* (Tomes, 1856) species complex is one such example that has long been taxonomically confused in the literature. *Crocidura watasei* (Kuroda, 1924), *C. tadae* (Tokuda et Kano, 1936), *C. kurodai* (Jameson et Jones, 1977), *C. wuchihensis* (Wang, 1966), and *C. indochinensis* (Robinson et Kloss, 1922) have been suggested to be junior synonyms or subspecies of *C. horsfieldii* (Corbet and Hill 1992; Hutterer 1993). However, subsequent researchers have re-evaluated the species status of these taxa (Hattori et al. 1990; Motokawa et al. 1996; Fang et al. 1997; Motokawa 1999; Fang and Lee 2002; Lunde et al. 2003, 2004; Chen et al. 2020). Among these species, the karyotypes of *C. watasei* (2n = 26), *C. tadae* (2n = 40), and *C. kurodai* (2n = 40) differ considerably from that of *C. horsfieldii* (2n = 38), providing further evidence for their recognition as distinct species (Krishna Rao and Aswathanarayana 1978; Tsuchiya et al. 1979; Harada et al. 1985; Fang et al. 1997; Biltueva et al. 2001; Fang and Lee 2002; Aswathanarayana 2003). However, no karyological information has yet been reported for *C. wuchihensis* and *C. indochinensis*.

Therefore, the present study aims to characterize the karyotype of *C. indochinensis* from Yunnan Province, southern China, using conventional and G-banding techniques, and to assess its karyotypic relationships with related *Crocidura* species.

## Material and methods

### Specimen

A female *Crocidura indochinensis* was captured using pitfall traps at an elevation of around 2,000 meters on Yongde Snow Mountain, Yunnan, China. The specimen was deposited in the Museum of Vertebrates, Kunming Institute of Zoology, Chinese Academy of Sciences, China. Species identification was based on detailed morphological examination and comparison with published taxonomic descriptions and diagnostic characters of *C. indochinensis*.

### Cell culture, metaphase preparation, and G-banding

Primary fibroblast cell lines were established from the ear and lung tissues of *C. indochinensis* using the explant culture method. In brief, small tissue fragments were cultured in DMEM medium supplemented with 15% fetal bovine serum, penicillin (100 U/mL) and streptomycin (100 µg/mL) at 37 °C and 5% CO_2_ for 3–4 weeks.

Metaphase chromosome preparations were obtained from primary fibroblast cultures using the standard technique. Prior to harvesting, the cells were treated with 10 µg/mL bromodeoxyuridine (BrdU, final concentration) for 12 h, followed by a medium replacement and further incubation for 3–4 h, and then treated with 0.05 µg/mL colchicine (final concentration) for approximately 1 h. Subsequently, the cells were treated with a hypotonic solution (0.075 mol/L KCl) for 15–20 min at room temperature or 37 °C, and then fixed twice in methanol/glacial acetic acid (3:1, v/v).

Chromosome spreads were prepared by applying 12 µL of metaphase suspension onto clean, dry slides, which were air-dried and subsequently placed in a ventilated oven at 65 °C for at least 4 hours. Conventional Giemsa staining was subsequently performed. GTG-banding was carried out using the trypsin/Giemsa staining method (Seabright 1972) with minor modifications. Chromosome spreads were treated with a 0.005% trypsin solution at room temperature for 5–7 min, rinsed in 2×SSC buffer and then stained with Giemsa stock solution diluted 1:6 in 0.025 M KH_2_PO_4_ buffer (pH 7.0) for 5 min.

### Microscopy and chromosome nomenclature

Digital images were captured and processed using the Genus System (Applied Imaging) with a Cohu CCD camera mounted on a Zeiss Axioplan 2 microscope, as previously described (Yang et al. 1999, 2006; Nie et al. 2002). The chromosomes of *C. indochinensis* were classified according to the system Levan et al. (1964).

## Results and discussion

**Figure 1.**
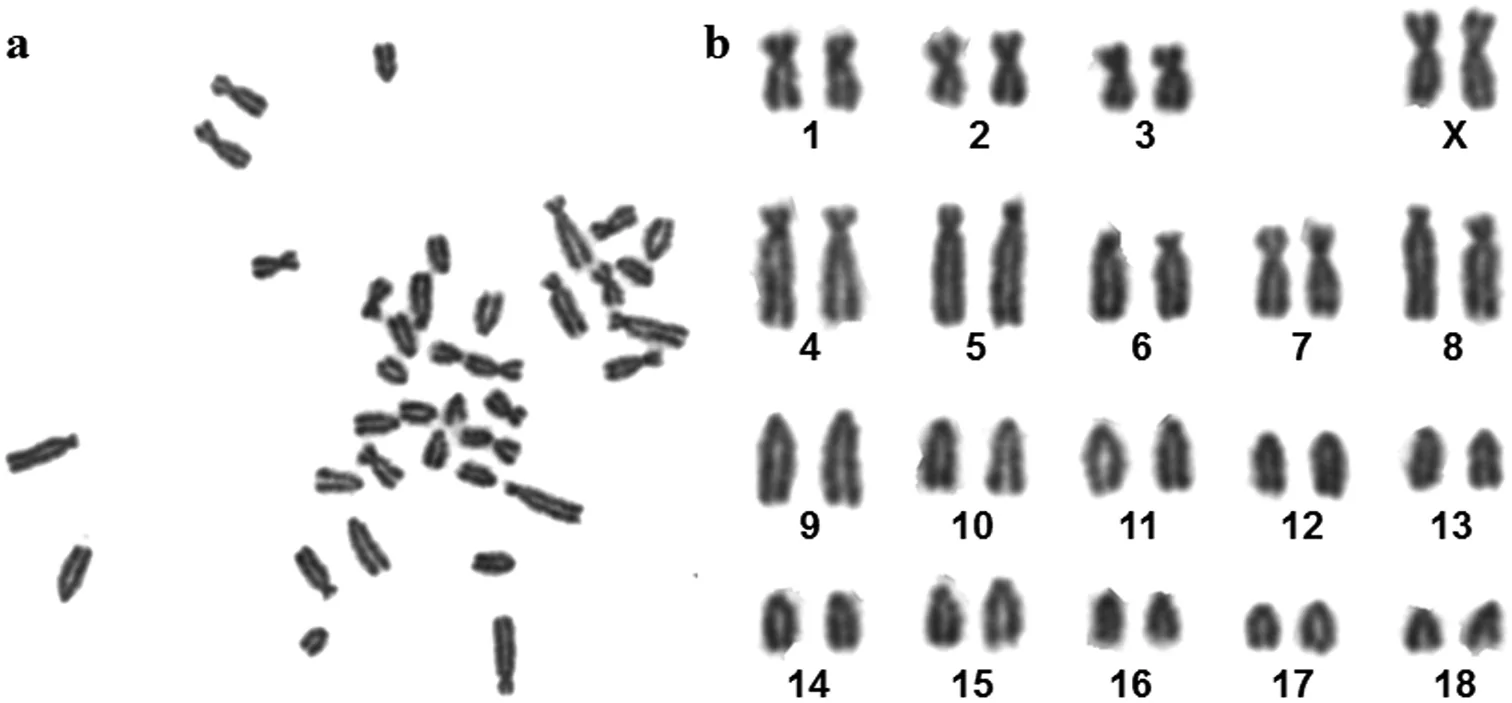
The karyotype of *Crocidura indochinensis* collected from Yongde Snow Mountain, Yunnan: **a**. Conventional metaphase plate; **b**. The karyotype.

**Figure 2.**
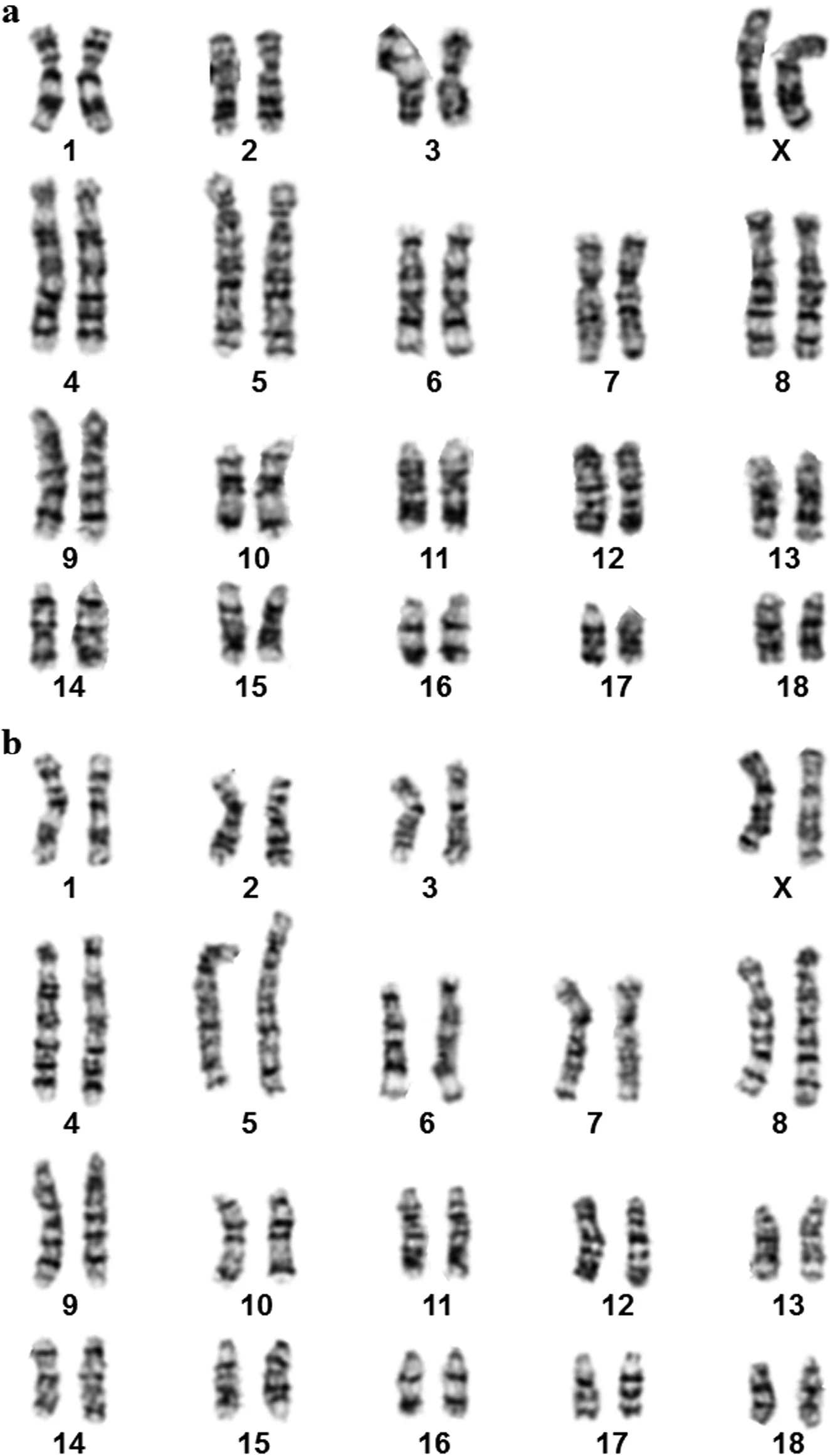
**a** and **b** G-banded karyotypes of *Crocidura indochinensis* from different metaphase spreads.

The conventional karyotype of *C. indochinensis* from Yunnan Province is reported here for the first time (Fig. 1). The diploid chromosome number (2n) and fundamental number (FN) of *C. indochinensis* were determined to be 38 and 56, respectively. The autosomes consisted of four metacentric/submetacentric pairs (nos. 1–3 and 7), four subtelocentric pairs (nos. 4–6 and 8), and ten acrocentric pairs (nos. 9–18). Although this karyotype was derived from a female specimen, comparisons with previously published G-banded karyotypes of male specimens from other Crocidurinae species indicated that the X chromosome was a medium-sized submetacentric chromosome. The GTG-banded karyotype of *C. indochinensis* is shown in Fig. 2, in which each chromosome pair displays a distinct G-banding pattern and homologous pairs are clearly identifiable.

Historically, *C. indochinensis* was regarded as a subspecies of *C. horsfieldii* by Ellerman and Morrison-Scott (1951), a classification subsequently adopted by several authors (Jenkins 1976; Heaney and Timm 1983; Hutterer 1993; Jiang and Hoffmann 2001). However, the two taxa exhibit markedly different biogeographical distributions. *C. indochinensis* occurs in northern and central Thailand, northern and southern Vietnam, northern and eastern Myanmar, northern and central Lao PDR, and southern China (Guizhou, Sichuan, and Yunnan provinces) (Hoffmann and Lunde 2008), whereas *C. horsfieldii* appears to be restricted to Sri Lanka and peninsular India (Lunde et al. 2003, 2004).

Our chromosomal analysis revealed that the karyotype of *C. indochinensis* (2n = 38, FN = 56) differs substantially from that reported for *C. horsfieldii* from peninsular India (2n = 38, FN = 48) (Krishna Rao and Aswathanarayana 1978), which comprises two meta- or submetacentric, two subtelocentric, and fourteen acrocentric autosomal pairs (Table 1). Therefore, the differences in biogeographical distribution and cytogenetic characteristics support the recognition of *C. indochinensis* as a distinct and valid species rather than a subspecies of *C. horsfieldii*.

**Table 1.** The karyotypic complement of species in the *Crocidura horsfieldii* complex. 2n = diploid number, FN = fundamental number, M = metacentric, SM = submetacentric, ST = subtelocentric, A = acrocentric.

**Species**	**Autosomes**			**Sex chromosomes**		**2n**	**FN**	**Types of cytogenetic staining**	**Speciemen origin**	**Reference**
	**M/SM**	**ST**	**A**	**X**	**Y**					
*C. horsfieldii*	2	2	14	M	SM	38	48	conventional Giemsa staining	India	Krishna Rao and Aswathanarayana 1978
*C. watasei*	10	2	–	SM	A	26	52	conventional Giemsa staining	Japan	Harada et al. 1985
								G-banding	Japan	Biltueva et al. 2001
*C. tadae*	3	3	13	SM	A	40	54	conventional Giemsa staining	Taiwan	Fang and Lee 2002
*C. kurodai*	3	3	13	SM	A	40	54	conventional Giemsa staining	Taiwan	Fang et al. 1997
*C. indochinensis*	4	4	10	SM	–	38	56	conventional Giemsa staining	Yunnan, China	Present paper
								G-banding		

Within the small-sized *Crocidura horsfieldii* complex, the karyotype of *C. indochinensis* (2n = 38, FN = 56) differs significantly from that of *C. watasei* (2n = 26, FN = 52) from Japan (Harada et al. 1985). The latter species is characterized by ten pairs of metacentric or submetacentric chromosomes, two pairs of subtelocentric chromosomes, a medium-sized submetacentric X chromosome, and a small acrocentric Y chromosome (Harada et al. 1985). The karyotype of *C. indochinensis* (2n = 38, FN = 56) also differs from those of *C. tadae* and *C. kurodai*, both of which are distributed in Taiwan and its offshore islands. These two species share the same karyotype (2n = 40, FN = 54), consisting of three pairs of metacentric or submetacentric chromosomes, three pairs of subtelocentric chromosomes, and thirteen pairs of acrocentric chromosomes, as well as a large submetacentric X chromosome and a small acrocentric Y chromosome (Fang et al. 1997; Fang and Lee 2002). Thus, these findings demonstrate that karyological data can provide valuable insights into the debated taxonomic status of *Crocidura* species.

**Figure 3.**
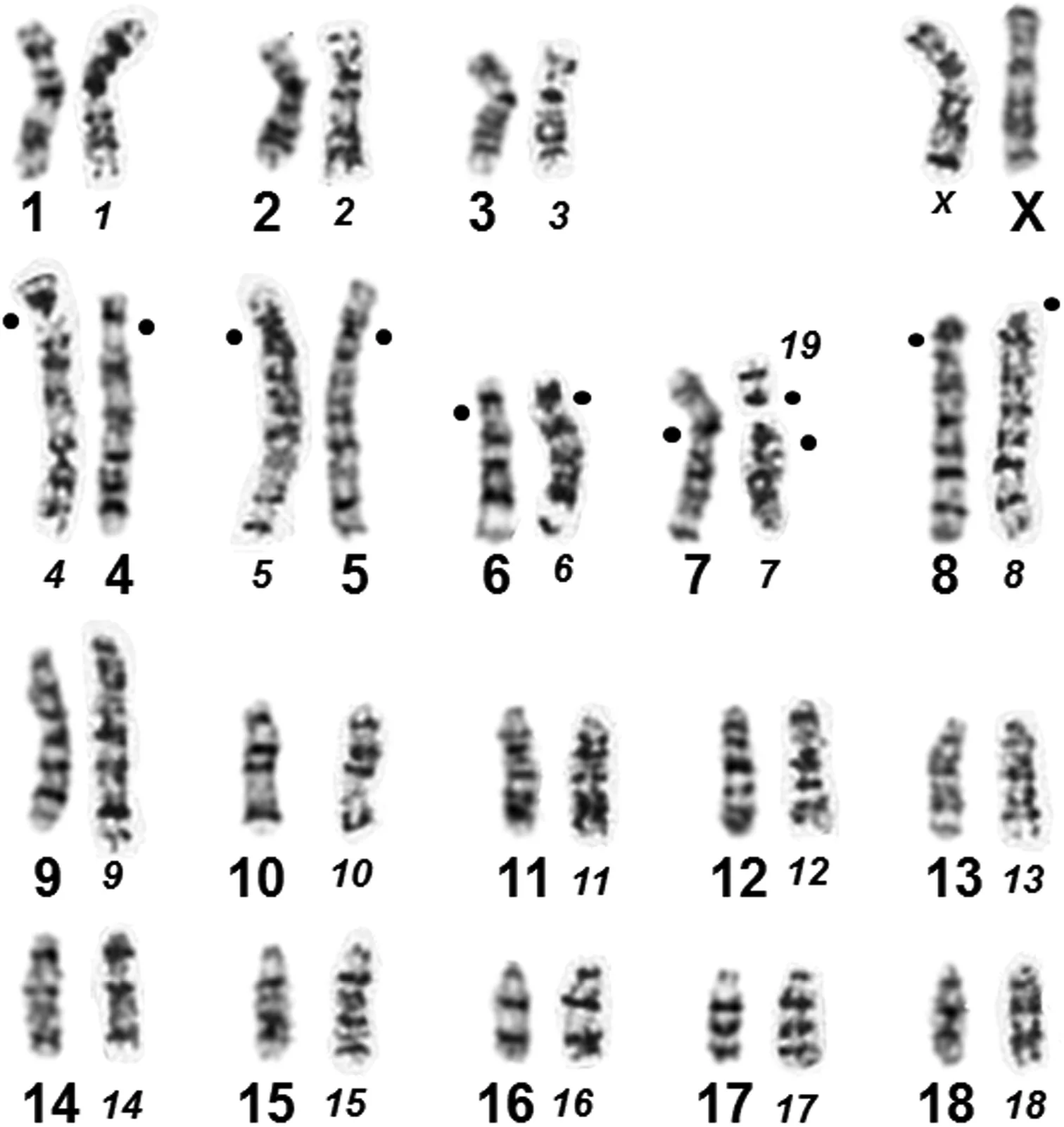
Comparison of G-banded haploid complements of *Crocidura indochinensis* (labeled with larger numbers) and *Crocidura dsinezumi* (smaller numbers in italics, taken from Fig. 1 of Biltueva et al. 2001). Dots indicate the centromeric position in certain chromosomes.

Biltueva et al. (2001) performed a comprehensive comparative analysis of high-resolution G-banded karyotypes in eight *Crocidura* species from the Palearctic and Oriental regions. Using the chromosomes of *C. dsinezumi* (2n = 40, FN = 56) as a reference, they revealed extensive chromosomal homology among these species. Accordingly, in the present study, we analysed the karyotypic relationship between *C. indochinensis* and *C. dsinezumi* to assess whether the two species exhibit highly conserved G-banding patterns (Fig. 3).

Sixteen of the eighteen autosomal pairs in *C. indochinensis* appear to have similar morphology and G-banding pattern to the corresponding pairs in *C. dsinezumi*. The metacentric, submetacentric, and subtelocentric chromosomes 1–6 in *C. indochinensis* are homologous to their counterparts in *C. dsinezumi*. Likewise, chromosomes 9–18, which are acrocentric, have a similar G-banding pattern in both species. The G-banding patterns of the proximal p-arm and the entire q-arm of submetacentric chromosome 7 in *C. indochinensis* corresponded to those of subtelocentric chromosome 7 in *C. dsinezumi*. However, the large distal region of the p-arm of chromosome 7 in *C. indochinensis* most likely corresponds to chromosome 19 of *C. dsinezumi*. Chromosome 8 is subtelocentric in *C. indochinensis* but acrocentric in *C. dsinezumi*. The observed differences in the banding pattern and centromeric position of chromosome 8 between the two species most likely resulted from a centromeric shift or a pericentric inversion. The X chromosomes of both species exhibited identical GTG-banding patterns. Overall, comparison of the G-banded karyotypes revealed a high degree of chromosomal homology between *C. indochinensis* and *C. dsinezumi* and identified a single fusion/fission event (*C. indochinensis* chromosome arms 7p and 7q correspond to *C. dsinezumi* chromosomes 19 and 7, respectively) and a centromeric shift or a pericentric inversion involving chromosome 8.

The karyotype of *C. indochinensis* (2n = 38, FN = 56) closely resembles the presumed ancestral karyotype of the genus *Crocidura* (2n = 38, FN = 54–58), which presumably consists of four pairs of metacentric chromosomes, three pairs of subtelocentric chromosomes, eleven pairs of acrocentric chromosomes, a metacentric X chromosome, and an acrocentric Y chromosome (Maddalena and Ruedi 1994). Furthermore, Biltueva et al. (2001, 2025) proposed that the putative ancestral karyotype of the genera *Crocidura*/*Suncus* had a diploid number of 2n = 44, comprising five pairs of meta- and submetacentric autosomes and 16 pairs of acrocentric autosomes, corresponding to *C. dsinezumi* chromosomes 1p, 1q, 2, 3, 4p, 4q, 5–13, 14prox, 14dist, 15, (16, 19), 17, and 18. Comparative analysis of G-banded karyotypes of *C. indochinensis* and *C. dsinezumi* showed that twelve of the eighteen autosomes of *C. indochinensis* (i.e., chromosomes 2, 3, 5, 6, 9–13, 15, 17, 18) have retained their ancestral state (Fig. 3). In contrast, chromosomes 1, 4, and 14 of *C. indochinensis* likely evolved through fusion events involving ancestral chromosomal segments homologous to *C. dsinezumi* chromosomes 1p and 1q, 4p and 4q, and 14prox and 14dist, respectively. The biarmed chromosome 7 of *C. indochinensis* was formed through a single break in the ancestral chromosome homologous to *C. dsinezumi* chromosomes 16 and 19, followed by fusion with the ancestral chromosome homologous to *C. dsinezumi* chromosome 7. The biarmed chromosome 8 of *C. indochinensis* differs from the ancestral chromosome homologous to the acrocentric chromosome 8 of *C. dsinezumi* by a centromeric shift or a pericentric inversion.

In summary, this study presents the first cytogenetic characterization of *C. indochinensis*. Comparative analysis of G-banded karyotypes revealed a high degree of chromosomal homology between *C. indochinensis* and *C. dsinezumi*, indicating a close karyotypic relationship between the two species. Their karyotypes differ in one fusion-fission event and one change in centromeric position. These findings enhance our understanding of karyotype evolution within the genus *Crocidura* and provide a basis for future cytogenetic, genomic, and evolutionary studies of this diverse group.

## Additional information

### Artificial Intelligence (AI) use

The authors accept full responsibility for the content of the manuscript, including the disclosure of any use of AI.

No AI tools were used in the preparation of this manuscript.

### Author ORCIDs

J. Ye https://orcid.org/0009-0002-4360-7876

### Data availability

All of the data that support the findings of this study are available in the main text.

## References

[B1] Aswathanarayana NV (2003) Karyotype evolution in the shrews, *Crocidura* and *Suncus* (Soricidae, Insectivora). Cytologia 68(1): 83–87. 10.1508/cytologia.68.83

[B2] Biltueva LS, Lemskaya NA, Abramov AV, Platonov VV, Serdyukova NA (2025) Comparative analysis of karyotypes of white-toothed shrews *Crocidura lasiura* and *Crocidura shantungensis* (Eulipotyphla, Mammalia). Mammalian Biology 106(3): 515–524. 10.1007/s42991-025-00548-1

[B3] Biltueva LS, Rogatcheva MB, Perelman PL, Borodin PM, Oda SI, Koyasu K, Harada M, Zima J, Graphodatsky AS (2001) Chromosomal phylogeny of certain shrews of the genera *Crocidura* and *Suncus* (Insectivora). Journal of Zoological Systematics and Evolutionary Research 39: 69–76. 10.1046/j.1439-0469.2001.00157.x

[B4] Burgin CJ, Colella JP, Kahn PL, Upham NS (2018) How many species of mammals are there? Journal of Mammalogy 99(1): 1–11. 10.1093/jmammal/gyx147

[B5] Chen S, Qing J, Liu Z, Liu Y, Tang M, Murphy RW, Pu Y, Wang X, Tang K, Guo K, Jiang X, Liu S (2020) Multilocus phylogeny and cryptic diversity of white-toothed shrews (Mammalia, Eulipotyphla, *Crocidura*) in China. BMC Evolutionary Biology 20(1): 29. 10.1186/s12862-020-1588-8 PMC7023792 32059644

[B6] Corbet GB, Hill JE (1992) The mammals of the Indomalayan region: a systematic review. Oxford University Press, New York, 488 pp. 10.5281/zenodo.13417191

[B7] Ellerman JR, Morrison-Scott TCS (1951) Checklist of Palaeartic and Indian mammals 1758 to 1946. Trustees of the British Museum (Natural History), London, 76 pp.

[B8] Fang YP, Lee LL, Yew FH, Yu HT (1997) Systematics of white-toothed shrews (*Crocidura*) (Mammalia: Insectivora: Soricidae) of Taiwan: karyological and morphological studies. Journal of Zoology (London) 242(1): 151–166. 10.1111/j.1469-7998.1997.tb02936.x

[B9] Fang YP, Lee LL (2002) Re-evaluation of the Taiwanese white-toothed shrew, *Crocidura tadae* Tokuda and Kano, 1936 (Insectivora: Soricidae) from Taiwan and two offshore islands. Journal of Zoology 257(2): 145–154. 10.1017/S0952836902000742

[B10] Harada M, Yoshida TH, Hattori S, Takada S (1985) Cytogenetical studies on Insectivora: III Karyotype comparison of two *Crocidura* species in Japan. Proceedings of the Japan Academy, Series B 61(8): 371–374. 10.2183/pjab.61.371

[B11] Hattori S, Yoshiyuki M, Noboru Y, Asaki H (1990) Geographical and ecological distributions and morphological variations of *Crocidura watasei* Kuroda, 1924 from the Amami Islands. Memoirs of the National Science Museum 23: 167–172. [In Japanese]

[B12] Heaney LR, Timm RM (1983) Systematics and distribution of shrews of the genus *Crocidura* (Mammalia: Insectivora) in Vietnam. Proceedings of the Biological Society of Washington 96: 115–120.

[B13] Hoffmann RS, Lunde D (2008) Soricomorpha. In: Smith AT, Xie Y (Eds) A guide to the mammals of China. Princeton University Press, New Jersey, 297–327. 10.1515/9781400834112

[B14] Hutterer R (1993) Order Insectivora. In: Wilson DE, Reeder DAM (Eds) Mammal species of the world: a taxonomic and geographic reference. Smithsonian Institution Press, Washington, 69–130.

[B15] Hutterer R (2005) Order Soricomorpha. In: Wilson DE, Reeder DAM (Eds) Mammal species of the world: a taxonomic and geographic reference. John Hopkins University Press, Baltimore, 220–311.

[B16] Jenkins PD (1976) Variation in Eurasian shrews of the genus *Crocidura* (Insectivora: Soricidae). Bulletin of the British Museum (Natural History) Zoology 30: 271–309. 10.5962/bhl.part.2386

[B17] Jiang XL, Hoffmann RS (2001) A revision of the white-toothed shrews (*Crocidura*) of southern China. Journal of Mammalogy 82(4): 1059–1079. 10.1644/1545-1542(2001)082<1059:AROTWT>2.0.CO;2

[B18] Krishna Rao S, Aswathanarayana NV (1978) Karyological analysis of the Horsfield’s shrew from peninsular India. Journal of Heredity 69(3): 202–204. 10.1093/oxfordjournals.jhered.a108927 731010

[B19] Levan A, Fredga K, Sandberg AA (1964) Nomenclature for centromeric position on chromosomes. Hereditas 52(2): 201–220. 10.1111/j.1601-5223.1964.tb01953.x

[B20] Lunde DP, Musser GG, Son NT (2003) A survey of the small mammals from Mt. Tay Con Linh II, Vietnam, with the description of a new species of *Chodsigoa* (Insectivora: Soricidae). Mammal Study 28(1): 31–46. 10.3106/mammalstudy.28.31

[B21] Lunde DP, Musser GG, Ziegler T (2004) Description of a new species of *Crocidura* (Soricomorpha: Soricidae, Crocidurinae) from Ke Go Nature Reserve, Vietnam. Mammal Study 29(1): 27–36. 10.3106/mammalstudy.29.27

[B22] Maddalena T (1990) Systematics and biogeography of Afrotropical and Palearctic shrews of the genus *Crocidura* (Insectivora: Soricidae) an electrophoretic approach. In: Peter G, Hutterer R (Eds) Vertebrates in the tropics. Museum Alexander Koenig Press, Bonn De, 297–308.

[B23] Maddalena T, Ruedi M (1994) Chromosomal evolution in the genus *Crocidura* (Insectivora: Soricidae). In: Merritt JF, Kirkland GL, Rose RK (Eds) Advances in Biology of Shrews. Carnegie Museum of Natural History Press, Pittsburgh, 335–344.

[B24] Motokawa M, Hattori S, Ota H, Hikida T (1996) Geographic Variation in the Watase’s shrew *Crocidura watasei* (Insectivora, Soricidae) from the Ryukyu Archipelago, Japan. Mammalia 60(20): 243–254. 10.1515/mamm.1996.60.2.243

[B25] Motokawa M (1999) Taxonomic history of the genus *Crocidura* (Insectivora: Soricidae) from Japan. In: Yokohata Y, Nakamura S (Eds) Recent Advances in the Biology of Japanese Insectivora – Proceedings of the Symposium on the Biology of Insectivores in Japan and on the Wildlife Conservation. Hiba Society of Natural History Press, Shobara, 63–71.

[B26] Nie W, Wang J, O’Brien PC, Fu B, Ying T, Ferguson-Smith MA, Yang F (2002) The genome phylogeny of domestic cat, red panda and five mustelid species revealed by comparative chromosome painting and G-banding. Chromosome Research 10: 209–222. 10.1023/A:1015292005631 12067210

[B27] Ruedi M, Vogel P (1995) Chromosomal evolution and zoogeographic origin of southeast Asian shrews (genus *Crocidura*). Experientia 51(2): 174–178. 10.1007/BF01929365 7875257

[B28] Seabright M (1972) The use of proteolytic enzymes for the mapping of structural rearrangements in the chromosome of man. Chromosoma 36: 204–210. 10.1007/BF00285214 5016007

[B29] Schlitter DA, Hutterer R, Maddalena T, Robbins LW (1999) New karyotypes of shrews (Mammalia: Soricidae) from Cameroon and Somalia. Annals of Carnegie Museum 68(1): 1–14. 10.5962/p.226615

[B30] Tsuchiya K, Yosida TH, Moriwaki K, Ohtani S, Kulta-Uthai S, Sudto P (1979) Karyotype of twelve species of small mammals from Thailand. Reports of the Hokkaido Institute of Public Health 29: 26–29. [In Japanese]

[B31] Yang F, O’Brien PC, Milne BS, Graphodatsky AS, Solanky N, Trifonov V, Rens W, Sargan D, Ferguson-Smith MA (1999) A complete comparative chromosome map for the dog, red fox, and human and its integration with canine genetic maps. Genomics 62(2): 189–202. 10.1006/geno.1999.5989 10610712

[B32] Yang F, Graphodatsky AS, Li T, Fu B, Dobigny G, Wang J, Li T, Fu B, Dobigny G, Perelman PL, Serdukova NA, Su W, O’Brien PC, Wang Y, Ferguson-Smith MA, Volobouev V, Nie W (2006) Comparative genome maps of the pangolin, hedgehog, sloth, anteater and human revealed by cross-species chromosome painting: further insight into the ancestral karyotype and genome evolution of eutherian mammals. Chromosome Research 14: 283–296. 10.1007/s10577-006-1045-6 16628499

[B33] Zima J, Lukáčová L, Macholán M (1998) Chromosomal evolution in shrews. In: Wójcik JM, Wolsan M (Eds) Evolution of Shrews. Mammal Research Institute Press, Białowieża, 175–218.

